# Initial and delayed thyroid-stimulating hormone elevation in extremely low-birth-weight infants

**DOI:** 10.1186/s12887-019-1730-1

**Published:** 2019-10-11

**Authors:** Shin Ae Yoon, Yun Sil Chang, So Yoon Ahn, Se In Sung, Won Soon Park

**Affiliations:** 1 Department of Pediatrics, Chungbuk National University Hospital, 1 Sunhwan-ro 776, Seowon-gu, Cheongju, 28644 South Korea; 2 Department of Pediatrics, Samsung Medical Center, Sungkyunkwan University School of Medicine, 81 Irwon-Ro, Gangnam-gu, Seoul, 06351 South Korea

**Keywords:** Thyroid function tests, Thyroxine supplementation, Preterm infants, Hypothalamic–pituitary–thyroid axis, Transient hypothyroxinemia of prematurity

## Abstract

**Background:**

To determine the incidence, etiology, and outcomes of thyroid-stimulating hormone (TSH) elevation in extremely low-birth-weight infants (ELBWIs).

**Methods:**

Newborn thyroid screening data of 584 ELBWIs (birth weight, < 1000 g; gestational age, ≥ 23 weeks) were retrospectively analyzed to identify initial (≤ 2 postnatal weeks) and delayed (> 2 weeks) TSH elevations. Growth and neurodevelopmental outcomes at 2 years’ corrected age (CA) were assessed according to levothyroxine replacement.

**Results:**

Initial and delayed TSH elevations were detected at CAs of 27 and 30 weeks, respectively, with incidence rates of 0.9 and 7.2%, respectively. All infants with initial TSH elevations had perinatal asphyxia, and 95% of those with delayed TSH elevation were exposed to various stressors, including respiratory support, drugs, and surgery within 2 weeks before diagnosis of TSH elevation. Free thyroxine (T4) levels were simultaneously reduced in 80 and 57% of infants with initial and delayed TSH elevations, respectively. Both initial and delayed TSH elevations were transient, regardless of levothyroxine replacement. Infants receiving levothyroxine replacement therapy had significantly higher TSH elevations, significantly lower free T4 levels, and significantly reduced mortality, compared to untreated infants. However, levothyroxine replacement had no significant effect on long-term growth and neurodevelopmental outcomes.

**Conclusions:**

The timing of insult superimposition on hypothalamic–pituitary–thyroid axis maturation is a major determinant of initial or delayed TSH elevation in ELBWIs. Levothyroxine replacement did not affect growth or neurodevelopmental outcomes in this population.

## Background

Thyroid-stimulating hormone (TSH) level is the preferred screening marker for the identification of infants with congenital hypothyroidism. Hypothyroidism in term newborn infants can easily be identified by an elevated TSH level together with a reduced thyroxine (T4) level after the second postnatal day [1]. However, it remains difficult to interpret TSH levels in extremely low-birth-weight infants (ELBWIs), with birth weights < 1000 g. Transient hypothyroxinemia of prematurity (THOP), defined as a temporarily low T4 level with a normal or low TSH level, is the most common thyroid dysfunction affecting preterm infants [2]. The etiology of THOP is considered to be multifactorial [2] and may include the postnatal cessation of maternal placental T4 transfer [3, 4], hypothalamic pituitary thyroid axis immaturity [5, 6], and non-thyroidal illness (NTI) [7–10]. Nonetheless, initial and delayed TSH elevations occur more frequently in preterm infants, especially ELBWIs, than in term infants [11, 12].

A recent study suggested that, besides primary congenital hypothyroidism, elevated TSH levels in preterm infants (< 28 weeks of gestation) might be associated with previous or concomitant inflammation, similarly to the non-thyroidal inflammation syndrome [13]. However, given the paucity of relevant clinical information, many factors remain unclear, including the true incidence of initial and delayed TSH elevation among ELBWIs, the clinical association of this condition with NTI, and the ability of thyroxine supplementation to improve outcomes in these cases. Therefore, the present retrospective observational study primarily aimed to determine the incidence of initial and delayed TSH elevation in ELBWIs. Secondarily, this study aimed to assess the clinical association of TSH elevation with NTI and to examine the effect of thyroxine supplementation on the growth, neurodevelopmental, and endocrine outcomes of ELBWIs at the corrected age (CA) of 2 years.

## Methods

We retrospectively reviewed the medical records of 584 ELBWIs (birth weights < 1000 g) with gestational ages (GAs) of ≥23 weeks who were born at and admitted to the Samsung Medical Center neonatal intensive care unit between January 2000 and July 2013, and for whom the results of initial thyroid function tests (TFTs) performed within the first 2 postnatal weeks were available. The data collection procedure was approved by the Institutional Review Board of Samsung Medical Center (2015–01-088), which waived the requirement for informed consent in this retrospective chart review.

Assays for TSH, T4, and free T4 (fT4) were performed using the Siemens ADVIA Centaur® XP chemiluminescent competitive immunoassay kits (Tarrytown, NY, USA). In this study, initial TSH elevation was arbitrarily defined as a TSH level > 20.0 μIU/ml [14, 15] during the initial TFT screening within the first 2 postnatal weeks of life. Delayed TSH elevation was defined as a TSH level ≤ 20.0 μIU/ml, regardless of the T4 level, during the same screening and a later TSH level increase > 20.0 μIU/ml [15] on the subsequent follow-up TFT. TFT normalization was defined as a decrease in the TSH level to 0.7–7.0 μIU/ml, the T4 level to 4.5–12.5 ng/dl, and the fT4 level to 0.9–1.8 ng/dl. Follow-up TFT screening tests were repeated every 2–6 weeks if the TFT results were abnormal and/or the infant was critically ill (including the requirement for mechanical ventilation or surgery) until a follow-up TFT yielded normalized results and the infant’s clinical condition stabilized. Levothyroxine supplementation for an initially relatively high (arbitrarily ≥40 μIU/ml) and/or sustained TSH elevation was initiated at a dosage of 10–15 μg/kg/d, based on recommendations from the attending pediatric endocrinologists.

Clinical characteristics, including GA, birth weight, Apgar scores at 1 and 5 min, sex, delivery mode, small for GA (birth weight below the 10th percentile), pregnancy-induced hypertension, gestational diabetes mellitus, and antenatal steroid use, were analyzed. GA was determined based on the maternal last menstrual period and the modified Ballard test. To identify the clinical risk factors associated with the development of TSH elevation, we investigated various confounding variables, including exposure to drugs such as dopamine and steroids, and surgery, especially 2 weeks prior to the detection of TSH elevation.

Outcome measures, including death before discharge, bronchopulmonary dysplasia (BPD) (≥ moderate) [16], intraventricular hemorrhage (IVH) (≥ grade 3) [17], periventricular leukomalacia (PVL), necrotizing enterocolitis (NEC) (≥ Bell’s stage 2b) [18], and retinopathy of prematurity (ROP) [19] requiring laser treatment, were also analyzed.

Upon follow-up at a CA of 2 years, each infant’s head circumference, height, and body weight were measured for the growth assessment. These values were converted to sex- and age-specific z scores of weight, height, and head circumference using the lambda, mu, and sigma method and the 2007 Korean National Growth Charts database [20]. Catch-up growth was defined as a weight, height, or head circumference exceeding the 10th percentile, according to the 2007 Korean National Growth Charts [20]. Neurodevelopmental factors such as cerebral palsy, hearing impairment, blindness, and Bayley scores were assessed. Cerebral palsy was defined as a Palisano gross motor function score ≥ 2. Blindness was defined as a visual acuity of < 20/200. Hearing loss was defined as bilateral impairment requiring hearing aids. Neurodevelopmental delay was defined as a mental developmental index or psychomotor developmental index score < 70, according to the Bayley Scales of Infant Development, Second Edition.

### Statistical analysis

Normally distributed continuous variables are expressed as means ± standard deviations. TSH levels were logarithmically transformed to assume a normal distribution and presented as geometric means. Comparisons of means were performed using the Student’s *t*-tests or the one-way ANOVA followed by multiple comparison tests using the Duncan’s method, while Mann–Whitney U test or Kruskal-Wallis test were conducted when variables are log transformed or a group has a sample size less than 30. Categorical variables are expressed as counts and percentages and were analyzed by the chi-square test or the Fisher’s exact test. Multivariate logistic regression was used to analyze the factors related to the clinical outcomes. Spearman’s correlation coefficients were calculated for the relationship between the fT4 and TSH levels. SPSS version 19.09 (SPSS Inc., Chicago, IL, USA) software was used for all statistical analyses, and a *P-value* < 0.05 was considered statistically significant.

## Results

In our study group, the mean GA and birth weight were 26 (range: 23–34) weeks and 769 ± 148 (370–999) g, respectively. The initial screening test was done at a mean of 7 ± 3 (1–14) postnatal days, and the second test was performed at a mean of 21 ± 17 (7–101) days. TFT screening was performed a mean of 3.7 ± 2.9 (1–10) times at mean intervals of 25 ± 8 (15–41) days during a mean hospital stay of 103 ± 52 (15–547) days. Of the 584 ELBWIs, 5 had initial TSH elevation. Of the 328 infants with initially lower T_4_ or fT_4_ levels and normal TSH levels, 39 developed delayed TSH elevation according to the subsequent TFT results. On the initial TFT, 251 patients had normal T_4_ and TSH levels; of these, 3 had delayed TSH elevations on the follow-up TFTs.

### Incidence of initial and delayed TSH elevation

**Fig. 1:**
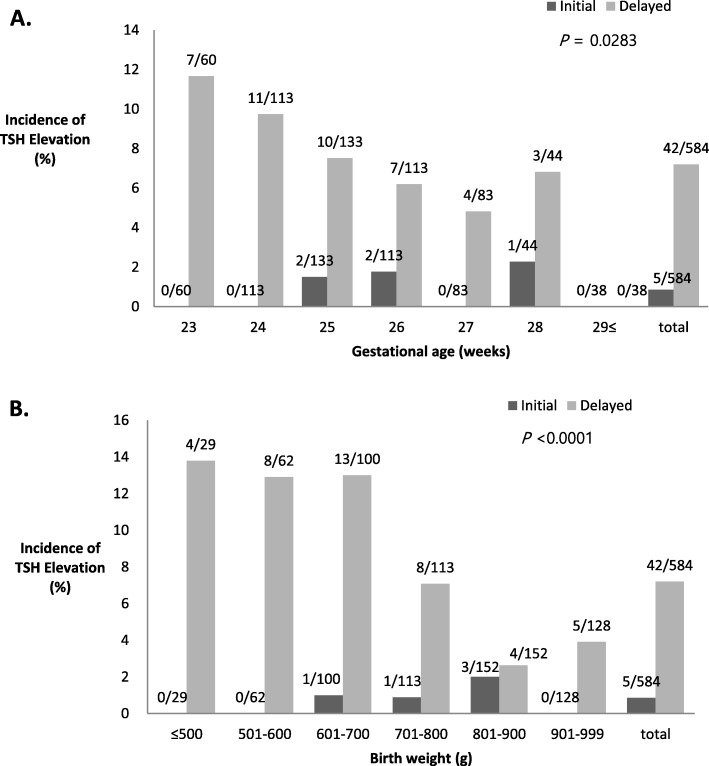
Incidence of initial and delayed thyroid-stimulating hormone elevations according to the gestational age (**a**) and birth weight (**b**)

The overall incidence rates of initial and delayed TSH elevation among ELBWIs were 0.9% (5/584) and 7.2% (42/584), respectively. The incidence rates of initial and delayed TSH elevation among ELBWIs according to GA and birth weight are shown in Fig. 1. Initial TSH elevation was observed only in infants with a GA of 25–28 weeks’ (1.3%, 5/373), and the highest incidence rate of 2.0% (3/152) was observed among infants with a birth weight in the 801–900 g range. However, the incidence of delayed TSH elevation exhibited significant inverse correlations with GA and birth weight, with the highest incidence rates of 11.7% (7/60) among infants at a GA of 23 weeks and 10.9% (33/304) among infants with birth weights ≤800 g.

### Clinical characteristics of infants with initial and delayed TSH elevations

The demographic and clinical characteristics of infants with initial and delayed TSH elevation are shown in Table 1. The Apgar score at 5 min and the frequency of antenatal steroid use were significantly lower among infants with initial TSH elevation, compared to those with delayed TSH elevation and normal controls. Statistically significant lower GA and birth weight were observed among infants with delayed TSH elevation, compared to those with initial TSH elevation and normal controls. No maternal thyroid disease or major congenital anomalies were observed.

**Table 1:** Demographics of infants with initial and delayed thyroid-stimulating hormone elevations and control

Variable	Initial TSH Elevation (*N* = 5)	Delayed TSH Elevation (*N* = 42)	Control (*N* = 251)
Gestational age (weeks)	26.0 ± 1.2	25.0 ± 1.5^*^	26.8 ± 1.9
	(25^+ 0^–28^+ 6^)	(23^+ 0^–28^+ 3^)	(24^+^ 0–34^+ 4^)
Birth weight (g)	789 ± 88	692 ± 142^*^	839 ± 120
	(648–884)	(470–990)	(380–999)
Male sex	3 (60)	15 (35)	108 (43)
One-minute Apgar score	3.0 ± 1.4	4.3 ± 1.7	4.8 ± 1.7
Five-minute Apgar score	5.6 ± 1.5^*, †^	7.2 ± 1.2	7.2 ± 1.4
Delivery type C/sec	5 (100)	32 (76)	206 (82)
Small for gestational age	1 (20)	10 (24)	66 (26)
Antenatal steroids	1 (20)^*, †^	36 (86)	200 (80)
Pathologic chorioamnionitis	1 (20)	13 (31)	116 (46)
Pregnancy-induced hypertension	3 (60)	7 (17)	62 (25)
Gestational diabetes mellitus	0 (0)	1 (2)	9 (4)

Values are presented as means ± SD (range) or n (%)

*TSH* Thyroid-stimulating hormone, *HD* Hospital day

^*^P < 0.05, vs Control

^†^*P* < 0.05, vs Delayed TSH elevation

### Clinical outcomes of infants with initial and delayed TSH elevations

The adverse clinical outcomes of infants with initial and delayed TSH elevations are shown in Table 2. The mortality rates of infants with initial TSH elevations tended to be higher than those of infants with delayed TSH elevations (*P* = 0.05) and normal controls (*P* = 0.028) after adjustment for GA, birth weight, Apgar score at 5 min and antenatal steroid use. However, there were no significant differences in the incidence rates of other morbidities such as BPD (≥ moderate), IVH (≥ 3), and PVL after adjusting for all the confounding variables.

**Table 2:** Clinical outcomes of infants with initial and delayed thyroid-stimulating hormone elevations and control

Outcome	Initial TSH Elevation (*N* = 5)	Delayed TSH Elevation (*N* = 42)	Control (*N* = 251)
Mortality	2 (40) ^*^	2 (5)	9 (4)
BPD (≥ moderate)	2/3 (67)	22/40 (55)	95/250 (38)
IVH (≥ 3)	1 (20)	7 (17)	13 (5)
PVL	1 (20)	3 (7)	15 (6)
NEC (≥ Stage 2b)	0 (0)	2 (5)	18 (7)
ROP requiring laser treatment	1/4 (25)	20 (48)	42/250 (17)
Composite morbidity	4 (80)	35 (83)	131 (52)

Values are presented as n (%). Values are adjusted by gestational age, birth weight, Apgar score at 5 min and antenatal steroid use

*BPD* bronchopulmonary dysplasia, *IVH* intraventricular hemorrhage, *PVL* periventricular leukomalacia, *NEC* necrotizing enterocolitis, *ROP* retinopathy of prematurity

^*^ *P* < 0.05, vs Control

### Thyroid function tests of infants with initial and delayed TSH elevation

**Fig. 2:**
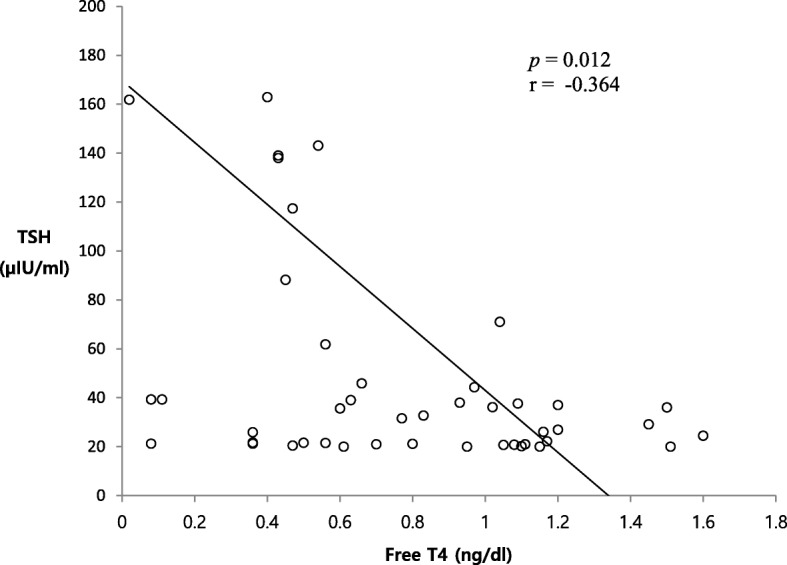
Spearman rank correlation analysis of thyroid-stimulating hormone and free thyroxine levels at the time of diagnosis of thyroid-stimulating hormone elevation

The TFT screening results are described in Table 3. The initial mean TSH level, measured at an average CA of 27.0 weeks, increased to a mean of 61.0 μIU/ml among infants with initial TSH elevation. Among infants with delayed TSH elevation, however, the initial TSH measured at an average CA of 26.0 weeks was normal, followed by a delayed increase to 78.3 μIU/ml at an average CA of 30.1 weeks. Whereas 80% (4/5) of infants with initial TSH elevation exhibited simultaneous reductions in T4 and/or fT4 levels, 93% (39/42) of infants with delayed TSH elevation exhibited decreased T4 and/or fT4 levels along with non-elevated TSH levels on the initial TFT. Moreover, the incidence of decreased T4 and/or fT4 levels was reduced to 57% (24/42) at the time of delayed TSH elevation detection. A Spearman correlation analysis revealed a significant inverse correlation of the extent of TSH elevation with the fT4 level (*r* = − 0.4, *P* = 0.012) (Fig. 2).

**Table 3:** Thyroid function test data of infants with initial and delayed thyroid-stimulating hormone elevations

		Initial TSH Elevation (*N* = 5)	Delayed TSH Elevation (*N* = 42)	*P* value
Initial TFT	Age, HD	8.6 ± 1.1 (7–10)	7.3 ± 2.0 (5–14)	0.15
	Corrected age	27.0 ± 1.6 (26^+ 3^–30^+ 1^)	26.0 ± 1.5 (24^+ 5^–29^+ 2^)	0.08
	T4 (μg/dl)	–	2.4 ± 2.0 (0.2–8.4)	–
	fT4 (ng/dl)^a^	0.7 ± 0.4 (0.1–1.1)	0.5 ± 0.0 (0.1–1.1)	–
	TSH (μIU/ml)	61.0 ± 81.9	3.3 ± 4.0	< 0.001
		36.7 (20.2–207.1)^b^	2.0 (0.4–17.4)^b^	0.001
Delayed TSH elevation	Age, HD	-	36.0 ± 25.2 (11–93)	-
	Corrected age	-	30.1 ± 3.9 (25^+ 3^–38^+ 1^)	-
	T4 (μg/dl)	–	2.4 ± 1.4 (0.2–6.5)	–
	fT4 (ng/dl)	–	0.7 ± 0.4 (0.02–1.6)	–
	TSH (μIU/ml)	–	78.3 ± 138.4	–
			35.3 (20.1–667.0)^b^	
	Peak TSH (μIU/ml)	–	88.1 ± 146.7	–
			37.3 (20.4–667.0)^b^	

*TSH* Thyroid-stimulating hormone, *TFT* Thyroid function test, *HD* Hospital day, *T4* Thyroxine, *fT4* free thyroxine

^a^The number of subjects with delayed TSH elevation was insufficient for comparison

^b^Geometric mean (range)

### Thyroid function tests with or without levothyroxine supplementation

Of the 47 infants with TSH elevation (5 initial, 42 delayed), 25 (1 initial, 24 delayed) received levothyroxine supplementation; the remaining 22 (4 initial, 18 delayed) received no treatment. In the non-treated group, one infant with an initial TSH elevation was declared NPO (nothing per oral) because of critical illness. The remaining 21 infants had relatively low TSH elevations (< 40 μIU/ml), with decreases in TSH levels at subsequent TFTs. At the initial TSH elevation, 1 infant had a very high TSH level of 207.1 μIU/ml and a low fT4 level and was treated with levothyroxine. Although the initial TFT results of infants with delayed TSH elevation did not differ significantly according to later levothyroxine supplementation, the TSH levels were significantly higher and fT4 levels significantly lower among infants with levothyroxine supplementation than among non-treated infants.

### Predisposing factors for the development of initial and delayed TSH elevation

All infants with an initial TSH elevation had perinatal asphyxia and remained intubated and ventilated, and 80% (4/5) were exposed to dopamine prior to the initial TFT performed at an average of 8.6 postnatal days. Among infants with delayed TSH elevation, 95% (40/42) had been exposed to more than one stressor, including respiratory support (37/42, among them, 28 were requiring ventilator support) and exposure to drugs (antibiotics: 32/42, dopamine 17/42, postnatal steroids: 13/42) and surgery (10/42), within 2 weeks prior to the detection of the delayed TSH elevation.

### Natural history and outcome of TSH elevation

Infants with TSH elevations were regularly followed up and either received no treatment or discontinued levothyroxine supplementation according to the decisions of the attending pediatric endocrinologists. TSH levels in the non-treated group normalized within a mean of 30.5 ± 21.3 days and were transient, except for 1 infant who died before the follow-up TFT evaluation. TSH levels in the treated group normalized within 21.8 ± 11.9 days and were transient as well. There was no significant difference in the time to normalize between the two groups. Infants in the treated group discontinued the levothyroxine supplementation with an average treatment duration of 635 ± 427 days. Their thyroid scan and sonogram revealed normal results, and the follow-up TFT remained normal up to 1 year after the discontinuation of the levothyroxine treatment.

### Short- and long-term outcomes according to levothyroxine supplementation

Regarding short-term outcomes, the incidence of morbidities such as BPD (≥ moderate), IVH (≥ 3), or PVL did not differ significantly by levothyroxine supplementation status. Although the incidence of mortality was significantly higher among non-treated infants than among levothyroxine-supplemented infants, this was attributed to underlying disease, rather than a lack of thyroid supplementation. A comparison of the long-term growth and neurodevelopmental outcomes of 17 out of 22 non-treated infants and 22 out of 25 levothyroxine-supplemented infants at a CA of 2 years revealed no significant differences in body weight, height, head circumference z scores, catch-up growth, the incidence of cerebral palsy, and neurodevelopmental delays.

## Discussion

In the present study, TSH elevation was detected in only 0.9% of ELBWIs during the initial TFT performed within 2 postnatal weeks, whereas delayed TSH elevation was detected in a significantly higher number of infants (7.2%). This relatively high incidence of initial TSH elevation among ELBWIs within 2 postnatal weeks is inconsistent with the current belief that initial TSH elevation is very rare [21–23]. Moreover, the finding of a 7.2% incidence of delayed TSH elevation in our cohort was much higher than the previously reported incidence of 1.7% among ELBWIs [11]. In the present study, the incidence of delayed TSH elevation correlated inversely with the GA and birth weight, even among these ELBWIs. Therefore, in addition to more diligent follow-up TFT screening, this wide discrepancy in the incidence of delayed TSH elevation might be attributable to disparities in the composition of the most immature population within these ELBWIs, characterized by GAs of 23–24 weeks and birth weights ≤800 g. These findings suggest that a modification to the TFT screening protocol, such as the establishment of a new routine of regular follow-up TFT screening program at 4 weeks intervals or until the patient becomes clinically stable, might be needed to detect hypothyroidism in extremely preterm infants near the limit of viability (i.e., GA of 23–24 weeks), who have the highest risk of developing delayed TSH elevation [24].

The etiology of initial and delayed TSH elevation in ELBWIs remains unclear. ELBWIs are more susceptible to morbidities, including perinatal asphyxia, infection, surgery, and exposure to thyroid function-inhibiting drugs, which could result in THOP [25, 26]. Moreover, as the hypothalamic–pituitary–thyroid axis has not been established in extremely preterm infants, the thyroid gland cannot generate sufficient levels of thyroid hormones in response to these stressors [27–29]. Therefore, the observation of THOP in critically ill ELBWIs might represent an epiphenomenon of these morbidities and could thus be considered an NTI [25, 30, 31]. In the present study, all infants with initial TSH elevations had perinatal asphyxia and remained intubated and ventilated, and 80% were exposed to dopamine despite its known suppression of TSH secretion [32] prior to the initial TFT (performed at a mean of 8.6 postnatal days). Furthermore, the frequency of antenatal steroid use and Apgar scores at 5 min were significantly lower among infants with initial TSH elevation, compared to those with delayed TSH elevation and normal controls. Although the initial TSH levels measured at a mean age of 7.3 postnatal days were normal, delayed TSH elevation was detected via follow-up TFTs performed at a mean of 36.0 postnatal days. Moreover, 95% of infants with delayed TSH elevation were exposed to multiple predisposing factors for TSH elevation, including respiratory support (88%), exposure to drugs such as dopamine (41%) and steroids (31%) despite their known inhibition of TSH secretion [32, 33], and surgery (24%), within 2 weeks prior to the detection of the delayed TSH elevation. In infants with delayed TSH elevation, the initial 93% incidence of decreased T4 and/or fT4 levels along with non-elevated TSH levels decreased to 57% when the delayed TSH elevation was detected. Overall, these findings suggest that the initial timing of insult determines whether TSH elevation is initial or delayed, and that both initial and delayed TSH elevations are representative of hypothyroidism caused by NTI during the early recovery phase and signal relief from the severity of the stressors [9, 34].

Critical changes in therapeutic strategy might have affected the clinical parameters, including the thyroidal function test results. During the study period, we experienced a decreased mortality and morbidity rate, as a result of better perinatal and neonatal intensive care, including the administration of more antenatal steroid use, active resuscitation at the delivery room, and application of a less-invasive ventilator management policy [35–37]. In parallel with the increased intact survival of ELBWIs, we observed significantly reduced incidences of THOP and subsequent TSH elevation in ELBWIs at 25–28 weeks’ gestation [38]. These findings support the assumption that the decrease in critical morbidities among ELBWIs due to the recent advances in neonatal intensive care medicine has decreased the incidence of TSH elevation in these infants.

Regarding the time of TSH elevation onset, initial TSH elevation was detected only in ELBWIs at a GA of 25–28 weeks, and was measured at a mean CA of 27 weeks. Although the highest incidence of delayed TSH elevation was observed in ELBWIs with a GA of 23 weeks, the initial TSH levels measured at a mean CA of 26 weeks were normal, whereas delayed elevation was indicated by a follow-up TFT performed at a mean CA of 30 weeks. These findings suggest that maturation of the hypothalamic–pituitary–thyroid axis [6, 27], when superimposed on the timing of insult, is the primary determinant of initial or delayed TSH elevation in an ELBWI.

The natural history of TSH elevation in ELBWI has not yet been delineated. In the present study, elevated TSH levels normalized at a mean age of 31 days, regardless of the levothyroxine supplementation status, and levothyroxine replacement therapy was discontinued at a mean age of 635 days. These findings support the idea that both the initial and delayed TSH elevations observed in ELBWIs represent epiphenomena of hypothyroidism due to NTI during the early recovery phase [13] and are therefore transient, in contrast to congenital hypothyroidism [39].

The indication for levothyroxine replacement therapy is another critical issue, requiring attention. The extent of thyroid dysfunction during NTI was found to correlate inversely to the severity of morbidity [25, 31, 40, 41]. In the present study, the extent of TSH elevation was found to exhibit a significant inverse correlation with the fT4 level. Furthermore, infants receiving levothyroxine replacement therapy had significantly higher TSH levels and significantly lower fT4 levels, compared with untreated infants. In our separate study [38], the initial fT4 level was the best predictor of mortality and composite morbidities compared with the Apgar and CRIB-II scores in ELBWIs. These findings suggest that although the levothyroxine replacement therapy was arbitrarily determined by the attending endocrinologist’s decision without clear treatment criteria, the initial critical morbidities experienced by infants receiving levothyroxine replacement therapy might have been more severe than those of untreated infants. Nevertheless, our data showing significantly improved mortality and no significant differences in growth and neurodevelopmental outcomes at a CA of 2 years, suggest that it might be prudent to administer levothyroxine to infants with high TSH and low fT4 levels. However, a high TSH level in the context of NTI is thought to signal relief from the severity of a stressor and has been associated with an improved prognosis in adults [42, 43]. Additionally, many clinical trials of premature infants failed to demonstrate the beneficial effects of levothyroxine replacement therapy [44–47]. Moreover, although our endocrinologists preferred longer treatment in the present study because thyroid hormones are critical for brain development during the first 2 to 3 years of life [48], the infants in a study by Woo et al. [11] received only a short-term levothyroxine replacement therapy. By contrast, the shortest duration of levothyroxine supplementation in the present study was 103 days. Additional well-designed studies are needed to determine the optimal indication, timing, and duration of levothyroxine replacement therapy.

The limitations of the present study include the single center, retrospective, and uncontrolled observational study design, as well as variations in the interval of the repeated TFT screening. Other potential confounders include variations in the indication, timing, and duration of levothyroxine replacement therapy, which depended entirely on the decisions of the attending endocrinologists. We also note that the small number of infants subjected to the Bayley tests was insufficient for an assessment of the long-term neurodevelopmental outcomes. Furthermore, we did not monitor iodine exposure, although we routinely used 0.5% chlorhexidine instead of iodine-containing agents as skin antiseptics during the procedures at our institute, throughout the study period. As only 24% of patients with delayed TSH elevation had undergone surgical procedures, the effect of iodine exposure on the incidence of TSH elevation might be insignificant.

## Conclusion

In conclusion, transient TSH elevation occurs frequently in ELBWIs and might result from NTI during the early recovery phase. The superimposition of the insult timing of stressors on the hypothalamic–pituitary–thyroid axis maturation is the primary determinant of initial or delayed TSH elevation. Additional well-designed prospective controlled studies are needed to clarify the benefits of levothyroxine replacement.

## Data Availability

The data that support the findings of this study are available from the corresponding author (wonspark@skku.edu) upon reasonable request.

## Abbreviations

BPD: Bronchopulmonary dysplasia
CA: Corrected age
ELBWI: Extremely low birth weight infant
fT4: Free thyroxine
GA: Gestational age
IVH: Intraventricular hemorrhage
MDI: Mental developmental index
NEC: Necrotizing enterocolitis
NTI: Non-thyroidal illness
PDI: Psychomotor developmental index
PVL: Periventricular leukomalacia
ROP: Retinopathy of prematurity
TFT: Thyroid function test
THOP: transient hypothyroxinemia of prematurity
TSH: thyroid-stimulating hormone
